# A Nonlinear Integrated Modeling Method of Extended Kalman Filter Based on Adaboost Algorithm

**DOI:** 10.3389/fchem.2021.716032

**Published:** 2021-07-30

**Authors:** Feng-Bo Zhou, Chang-Geng Li, Hong-Qiu Zhu

**Affiliations:** ^1^ School of Information Engineering, Shaoyang University, Shaoyang, China; ^2^ School of Physics and Electronics, Central South University, Changsha, China; ^3^ School of Automation, Central South University, Changsha, China

**Keywords:** zinc hydrometallurgy, extended Kalman filter spectrophotometry, ultraviolet visible spectroscopy, integrated modeling, Adaboost algorithm

## Abstract

Abstract In the zinc hydrometallurgical purification process, the concentration ratio of zinc ion to trace nickel ion is as high as 10^5^, so that the nickel spectral signal is completely covered by high concentration zinc signal, resulting in low sensitivity and nonlinear characteristics of nickel spectral signal. Aiming at the problem that it is difficult to detect nickel in zinc sulfate solution, this paper proposes a nonlinear integrated modeling method of extended Kalman filter based on Adaboost algorithm. First, a non-linear nickel model is established based on nickel standard solution. Second, an extended Kalman filter wavelength optimization method based on correlation coefficient is proposed to select wavelength variables with high signal sensitivity, large amount of information and strong nonlinear correlation. Finally, a nonlinear integrated modeling method based on Adaboost algorithm is proposed, which uses extended Kalman filter as a basic submodel, and realizes the stable detection of trace nickel through the weighted combination of multiple basic models. The results show that the average relative error of this method for detecting nickel is 4.56%, which achieves accurate detection of trace nickel in zinc sulfate solution.

## Introduction

Zinc (Zn) is the third largest non-ferrous metal after aluminum and copper (Yadav and Banerjee, 2018). At present, 80% of zinc in the world is produced by zinc hydrometallurgy (Zhou et al., 2019), which mainly includes grinding, leaching, purification and electrolysis processes (Zhang et al., 2018; Li et al., 2018). Among them, the purification process is a key process of zinc hydrometallurgy (Luo et al., 2019), through the accurate detection of impurity ion concentration in the electrolyte, and then based on the zinc powder replacement method to remove impurity ions, that is the premise to ensure the normal operation of zinc hydrometallurgy (Zahmatkesh, et al., 2017; Molognoni et al., 2018). However, manual offline analysis of the impurity ion concentration is still used in the current production (Kočanová et al., 2017). The detection is time-consuming (Chen et al., 2018), the process is cumbersome (Chen et al., 2017), and the feedback information is lagging (Dong et al., 2018), so it is impossible to optimize and adjust the process parameters of the zinc hydrometallurgical purification process in real time (He et al., 2016). Usually, excessive addition of zinc powder is used to ensure the impurity removal effect, resulting in large consumption of zinc powder (Li et al., 2017), large fluctuation of impurity ion concentration (Walash et al., 2009), low production efficiency and unqualified product quality (Zhou et al., 2009; Ji et al., 2012), which restrict the high-efficiency and green production of purification process (Liu et al., 2017). Therefore, it is urgent to realize the on-line detection of the concentration of impurity metal ions in zinc hydrometallurgy.

In recent years, the commonly used methods for on-line detection of metal ions include potentiometric titration (Attimarad 2012), polarography (Duran Meras et al., 2002), inductively coupled plasma mass spectrometry (Rastogi et al., 2015), ultraviolet visible spectroscopy (Lambert et al., 2017) and laser-induced breakdown spectroscopy (Fedenko et al., 2017). As a conventional quantitative analysis method, ultraviolet visible spectroscopy is widely used in the field of analysis and detection due to its advantages of simplicity, accuracy, rapidity, multi-function and low cost (Lepane et al., 2015; Lee and Oh, 2017). However, in the zinc hydrometallurgical purification process, when using ultraviolet-visible spectroscopy to detect nickel (Ni) in zinc sulfate solution, there are the following difficulties (Rote and Bari, 2009; Khan et al., 2020): 1) in zinc sulfate solution, the concentration ratio of main zinc to nickel ion is as high as 10^5^, which makes the nickel spectral signal seriously masked by the main zinc signal; 2) the chemical characteristics of zinc and nickel in zinc solution are similar, and the spectral characteristic information area is concentrated and the effective band is narrow, which makes their spectral signal seriously overlap and interfere with each other; 3) due to the increase of particle density in zinc sulfate solution, the absorptivity and transmissivity of the solution to the incident light are changed, resulting in the spectral characteristics of nickel in the solution seriously deviate from Beer-Lambert law, which makes it difficult to quantitatively analyze and detect nickel; 4) due to the fluctuation of the main zinc, the random noise of the testing instrument and the weak background interference of reagents such as chromogenic agents, these factors will affect the detection accuracy of nickel ion. The above-mentioned problems have brought great challenges to detecting the concentration of nickel ion in zinc sulfate solution based on ultraviolet-visible spectroscopy.

In order to solve the problem that it is difficult to detect the concentration of nickel ion in zinc sulfate solution, this paper proposes a nonlinear integrated modeling method of extended Kalman filter based on Adaboost algorithm. First, a non-linear nickel model is established based on nickel standard solution. Second, an extended Kalman filter wavelength optimization method based on correlation coefficient is proposed to select wavelength variables with high signal sensitivity, large amount of information and strong nonlinear correlation. Finally, a nonlinear integrated modeling method based on Adaboost algorithm is proposed, which uses extended Kalman filter as a basic submodel, and realizes the stable detection of trace nickel ion through the weighted combination of multiple basic models. The results show that the average relative error of this method for detecting nickel ion is 4.56%, which achieves accurate detection of trace nickel ion in high concentration zinc solution.

## Theory

### A. Extended Kalman Filter

Kalman filter spectrophotometry takes the minimum mean square error as the criterion to mathematically process a series of measurement data containing errors (Ben-Ari and Ben-shahar, 2013). It is suitable for the spectrophotometric analysis of multi-component systems and is currently mainly used for multi-component linear modeling (Ji et al., 2012). Aiming at the nonlinearity of nickel in high-concentration zinc solution, based on the Kalman filter spectrophotometry, this paper proposes an extended Kalman filter spectrophotometric method based on ultraviolet-visible spectroscopy to detect nickel ion in high concentration zinc solution. Generally, the extended Kalman filter (EKF) describe a nonlinear system using a system model and a measurement model, these two models can be expressed as follows:

X(k)=X(k−1)+W(k) (1)

Z(k)=h[X(k)]+V(k) (2)

where, k represents the wavelength, X(k) is the concentration, W(k) is the system noise, h[·] represents the non-linear absorption coefficient related to the concentration X(k), Z(k) represents the absorbance and V(k) represents the measurement noise.

By expanding Eq. 2 according to the Taylor series and omitting the terms of the second order and above, it is obtained that:

$$Z\left(k\right)=h\left[\overset{\wedge }{X}\left(k|k-1\right),k\right]+{\frac{\partial h}{\partial \overset{\wedge }{X}\left(k\right)}|}_{\overset{\wedge }{X}\left(k|k-1\right)}\left[X\left(k\right)-\overset{\wedge }{X}\left(k|k-1\right)\right]+V\left(k\right)$$ (3)

and set as

$$H\left(k\right)={\frac{\partial h}{\partial \overset{\wedge }{X}\left(k\right)}|}_{\overset{\wedge }{X}\left(k|k-1\right)}\;\;y\left(k\right)=h\left[\overset{\wedge }{X}\left(k|k-1\right),k\right]-{\frac{\partial h}{\partial \overset{\wedge }{X}\left(k\right)}|}_{\overset{\wedge }{X}\left(k|k-1\right)}\overset{\wedge }{X}\left(k|k-1\right)$$ (4)

From Eq. 3 and Eq. (4), the measurement model can be written as follows:

Z(k)=H(k)X(k)+y(k)+V(k) (5)

According to the linearization models of Eq. 1 and Eq. (5), the optimal estimate can be obtained from the previous estimate and the current measurement, and is given by:

$$\overset{\wedge }{X}\left(k\right)=\overset{\wedge }{X}\left(k-1\right)+K\left(\text{k}\right)\left[\text{Z}\left(k\right)-H^{T}\left(k\right)\cdot X\left(k-1\right)\right]$$ (6)

In Eq. 6, K(k) represents the Kalman gain matrix, given by

$$K\left(k\right)=P\left(k-1\right)H\left(k\right){\left[H^{T}\left(k\right)P\left(k-1\right)H\left(k\right)+R\left(k\right)\right]}^{-1}$$ (7)

where, R(k) is the measurement noise of the instrument and P(k−1) is the error covariance matrix, respectively expressed as follows:

$$R\left(k\right)=\frac{1}{m}\sum_{k=1}^{m}V\left(k-1\right)V\left(k-1\right)-H^{T}\left(k\right)P\left(k-1\right)H\left(k\right)$$ (8)

$$P\left(k\right)=\left[I-K\left(k\right)H^{T}\left(k\right)\right]P\left(k-1\right){\left[I-K\left(k\right)H^{T}\left(k\right)\right]}^{T}+K\left(k\right)R\left(k\right)K^{T}\left(k\right)$$ (9)

Eq. 1, Eq. 2, Eq. 3, Eq. 4, Eq. 5, Eq. 6, Eq. 7, Eq. 8, and Eq. 9 constitute the basic extended Kalman filter spectrophotometry.

### B. Integrated Modeling Based on Adaboost Algorithm

In zinc sulfate solution, the concentration ratio of zinc ion to nickel ion is as high as 10^5^, which leads to low sensitivity and strong nonlinearity of nickel spectral signal. The extended Kalman filter algorithm proposed above can better detect nickel concentration in zinc solution, but the relative error varies greatly. The main reasons for this problem are the fluctuation of the main zinc, the mutual interference and suppression between multi metal ions, the random noise of detection instrument and the weak background interference of reagents such as chromogenic reagent. These factors will affect nickel absorbance, thus affecting the detection accuracy and repeatability of a single sample, resulting in excessive sample difference.

**FIGURE 1:**
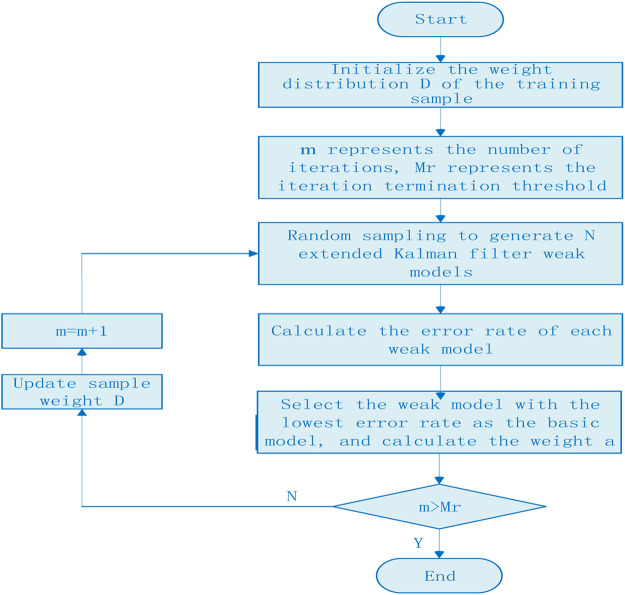
The flow chart of Adaboost training sample.

Integrated modeling is an effective method to solve the low stability of a single model. Its core idea is to form multiple training sets from a same training set randomly or in combination, then build sub-models based on these training sets, and use the sub-models to predict separately, and finally use the integrated method to form a final result. In this paper, the idea of integrated modeling is applied to the concentration detection of trace nickel in high-concentration zinc solution, and an integrated modeling method based on Adaboost algorithm is proposed. Taking extended Kalman filter as the basic sub-model, through the weighted combination of multiple basic models, the weight of weak classifiers with a large classification error rate is reduced, while the weight of weak classifiers with a small classification error rate is increased, so as to improve the sample detection accuracy, enhance the stability of the model, and realize the stable detection of trace nickel in zinc sulfate solution. The flow chart of Adaboost training sample is shown in Figure 1.

In the Adaboost algorithm, the weight distribution of training samples is initialized firstly. Assuming that there are a total of N training samples, each sample is assigned the same sample weight value 1/N, and these weight values constitute the vector D. The specific implementation steps are as follows:

1) Initialize the weight distribution of training samples:

$$D_{l}=\left\{w_{ll},\cdot \cdot \cdot w_{li},\cdot \cdot \cdot ,w_{lN}\right\},w_{li}=1/N,i=1,2,\cdot \cdot \cdot ,N$$ (10)

2) These samples are used to train the weak classifier, and the weight of samples is adjusted to train iteratively. m is the *m*th iteration, and the maximum number of iterations $M_{r}$ is set to 40.
3) According to the characteristic wavelength variables, several weak models of EKF are randomly generated. The weak model with the lowest error rate is selected as the *m*th basic model to predict the validation samples, and the maximum error rate and the relative error of each sample are calculated.

$$E_{m}=\max\left|y_{i}-G_{m}\left(x_{i}\right)\right|$$ (11)

$$\varepsilon_{mi}=\frac{\left|y_{i}-G_{m}\left(x_{i}\right)\right|}{E_{m}}$$ (12)

4) The total error rate of the *m*th basic model is calculated.

$$\varepsilon_{m}=\sum_{i=1}^{N}w_{mi}\varepsilon_{mi}$$ (13)

5) The weight of the *m*th weak classifier *H*_*m*_(*x*) in the final model is calculated.

$$\alpha_{m}=\frac{1}{2}\ln\left(\frac{1-\varepsilon_{m}}{\varepsilon_{m}}\right)sign\left(\frac{1}{2}\ln\left(\frac{1-\varepsilon_{m}}{\varepsilon_{m}}\right)\right)$$ (14)

According to Eq. 14, $\alpha_{m}$increases with the decrease of $\varepsilon_{m}$, which indicates that in the final classifier, the weight proportion of weak classifier with small classification error rate can be increased.

6) Updating the weight distribution of training set.

$$D_{m+1}=\left\{w_{m+1,l},\cdot \cdot \cdot w_{m+1,i},\cdot \cdot \cdot ,w_{m+1,N}\right\},i=1,2,\cdot \cdot \cdot ,N$$ (15)

$$w_{m+1,i}=\frac{w_{mi}\exp\left(-\alpha_{m}y_{i}\hat{y_{i}}\right)}{Z_{m}}$$ (16)

$$Z_{m}=2\sqrt{\varepsilon_{m}\left(1-\varepsilon_{m}\right)}$$ (17)

where, $y_{i}$ and $\hat{y_{i}}$ represent the calibration value and predicted value of the *i*th sample respectively, and $Z_{m}$ represents the normalization factor.

7) If the number of iterations exceeds the set threshold, the trained weak classifiers are combined into strong classifiers.

$$f\left(x\right)=sign\left(\sum_{k=1}^{K}\alpha_{k}G\left(x\right)\right)$$ (18)

## Experimental

### A. Reagents

All chemicals were analytical reagents and no further purification was required. Deionized water was used as solvent to prepare 50 g/L zinc and 12.5 mg/L nickel stock solutions. The standard solution was then prepared by continuous dilution of the stock solution as required. Acetic acid-sodium acetate buffer (pH = 5.5) was prepared by mixing an appropriate volume of pure acetic acid and sodium acetate. Ethylene Diamine Tetraacetic Acid (EDTA, 1.0 mol/L) was used as a masking solution to reduce the absorbance of zinc. Cetyltrimethylammonium bromide (CTAB, 0.01 mol/L) was used as a stabilizer solution. Nitroso-R salt solution (0.4%) was prepared as a chromogenic reagent.

### B. Apparatus

An Optosky ATP2000 micro spectrometer (Optosky Technology Ltd., China) was used to measure the absorbance spectra. Quartz cuvettes (1 cm) were matched and used for all absorbance measurements. ATP2000 is a high-sensitivity miniature optical fiber spectrometer. It adopts a 2048 × 64 pixel refrigerated linear back-illuminated CCD detector, which greatly reduces the noise of the sensor and improves the reliability of measurement. The detection spectral wavelength range is 180–1100 nm, the optical resolution is up to 0.1 nm, and the measured spectral data is output through USB2.0 or UART port.

### C. Procedures

In a 25 ml calibration flask, add different proportions of zinc and nickel standard solutions, 7.5 ml acetic acid-sodium acetate buffer solution (pH = 5.5) and 5.00 ml Nitroso R salt solution, and dilute to the calibration scale with an appropriate volume of distilled water. The blank solution was prepared in the same way. The final concentration range of zinc is 20–30 g/L, and that of nickel is 0.6–6.0 mg/L. The ATP-2000 spectrophotometer is used to measure the spectral signal of the mixed solution, scanning in the wavelength range of 350–800 nm at 1 nm interval. All measured spectra are the average of five repeated measurements.

## Results and Discussion

### A. Spectral Characteristics

**FIGURE 2:**
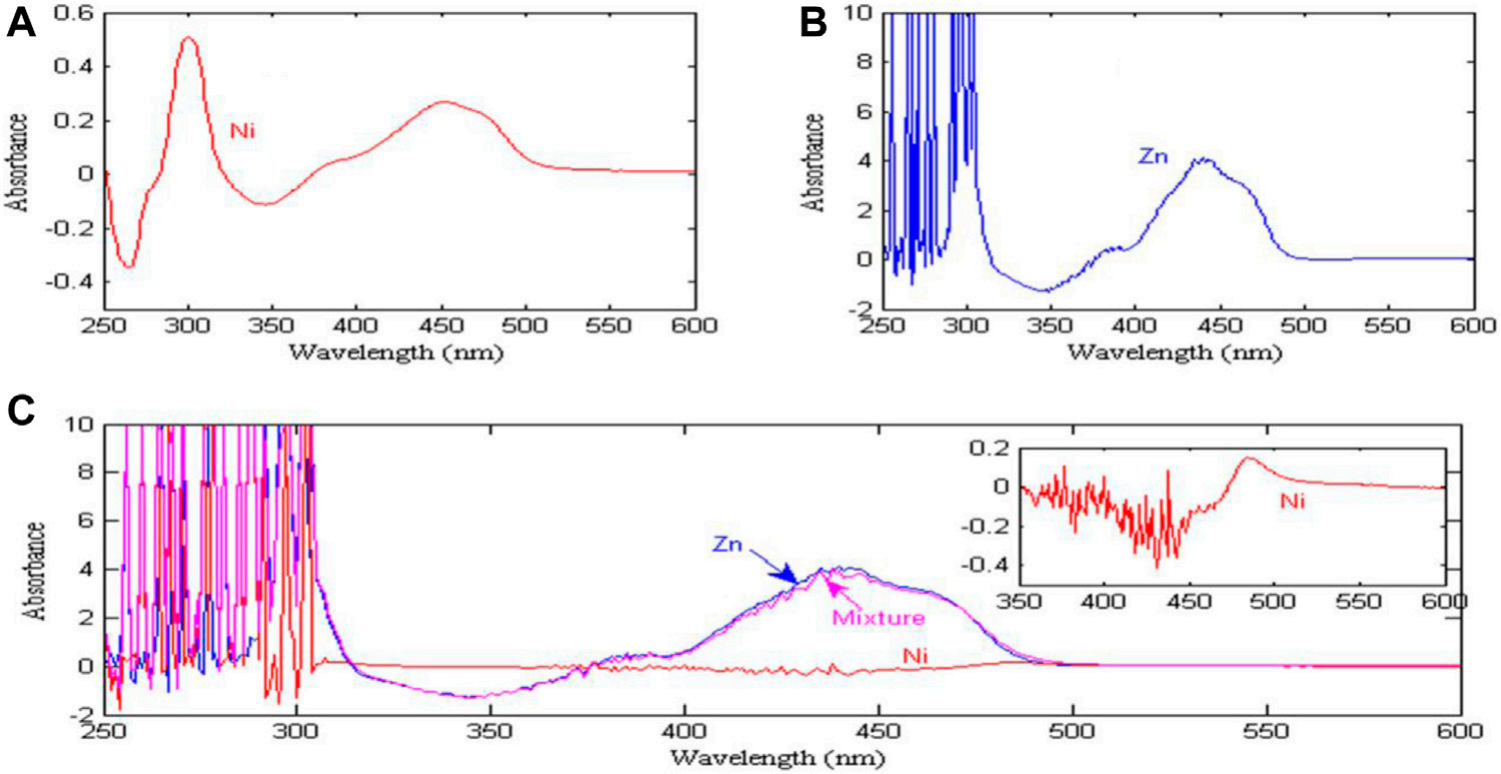
Absorption spectra of zinc and nickel. **(A)** Absorption spectra of pure nickel solution; **(B)** Absorption spectra of pure zinc solution: Show results in wavelength range from 375 nm to 600 nm and Absorbance range from 0 to 6 (never below 0); **(C)** Absorption spectra of zinc, nickel and their mixtures: Show results in wavelength range from 300 nm to 600 nm and Absorbance range from 0 to 6 (never below 0); Show results in wavelength range from 475 nm to 600 nm and Absorbance range from 0 to 0.2 (never below 0).

The basic principle of ultraviolet-visible absorption spectroscopy detection is Beer-Lambert law, that is, when a beam of parallel monochromatic light passes through a uniform colored solution, when the optical path is fixed, the absorbance of the solution is proportional to the concentration of the solution. Figure 2A shows the absorption spectral signal of a 0.6 mg/L nickel solution. There are characteristic peaks at wavelengths of 302 and 455 nm, and the maximum absorbance is 0.51. Figure 2B shows the spectral signal of a 20 g/L zinc solution, with a characteristic peak at a wavelength of 441 nm, and a maximum absorbance of 4.149. Figure 2C shows the spectral signal of nickel (0.6 mg/L), zinc (20 g/L) and their mixture in 250–600 nm. It can be seen from Figure 2C that the absorbance of zinc is close to the absorbance of its mixture, which almost completely masks the nickel signal, resulting in a lower absorbance of nickel. From the partial enlarged view of nickel signal, it can be seen that the characteristic peak of nickel is shifted due to the influence of high concentration zinc signal, and the characteristic peak appears at the wavelength of 486 nm, and the maximum absorbance is only 0.15. In 250–300 nm, due to the supersaturation of zinc ion, the measured absorbance exceeds the maximum range of the instrument, and the measured value is not available. In addition, because zinc and nickel have similar chemical properties, the absorption spectra of zinc and nickel are seriously overlapped in the entire wavelength range. Therefore, it is difficult to detect the concentration of nickel in high concentration zinc solution.

**FIGURE 3:**
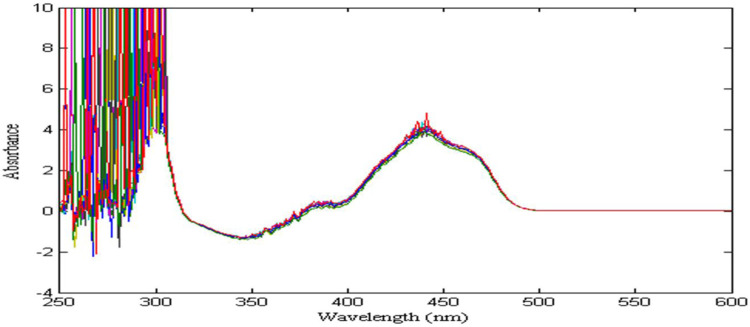
The absorption spectral signal of zinc solution in the range of 20–30 g/L: Show results in wavelength range from 390 nm to 600 nm and Absorbance range from 0 to 6 (never below 0).

The absorption spectra of zinc (20–30 g/L) are shown in Figure 3, it can be seen that as the zinc concentration increases, there is almost no change in absorbance. The reason is that the concentration of zinc is oversaturated, which leads to the relationship between its absorbance and concentration no longer obeys Beer-Lambert law. Therefore, in the detection of nickel concentration in zinc sulfate solution, the preparation of 20 g/L zinc solution as a reference solution can eliminate the interference of zinc on nickel signal in their mixed solution, and improve the sensitivity and resolution of nickel spectral signal.

### B. Univariate Calibration

**FIGURE 4:**
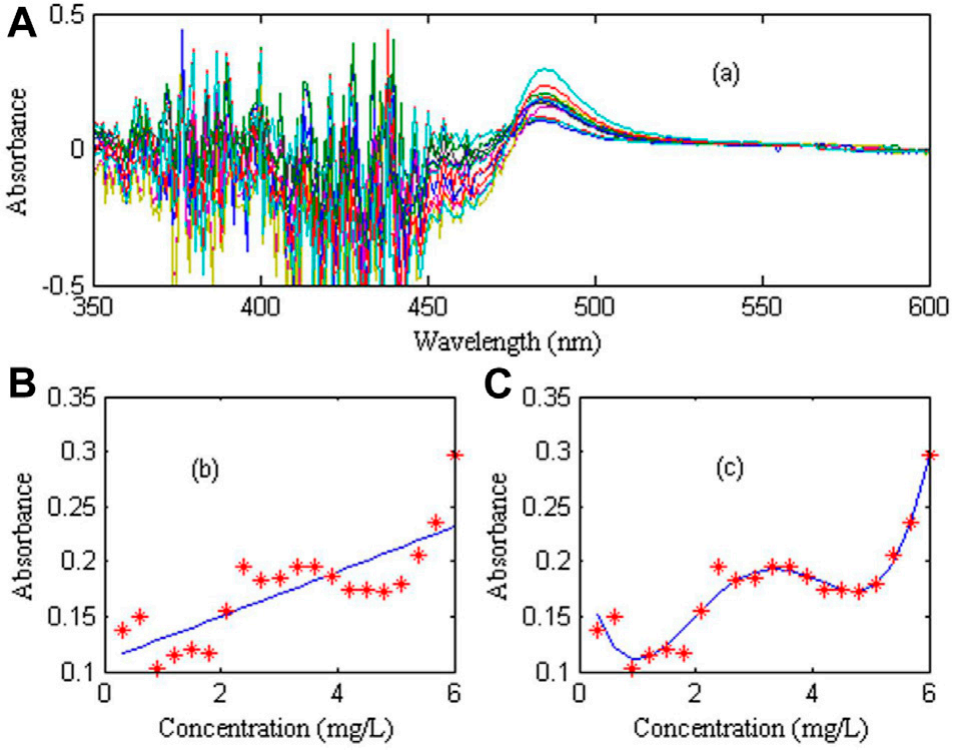
Calibration curve for nickel. **(A)** Absorption spectra of nickel standard solutions with different concentrations: Show results in wavelength range from 475 nm to 600 nm and Absorbance range from 0 to 6 (never below 0). **(B)** Linear calibration curve. **(C)** Nonlinear calibration curve.

In order to evaluate the linearity of trace nickel in zinc sulfate solution, 20 groups of 0.6–6.0 mg/L nickel standard solution and a 20 g/L zinc solution as reference were prepared. The calibration curve was constructed by using the absorbance of nickel at the maximum peak (wavelength 486 nm) and the corresponding concentration. The linear and nonlinear models were used for calibration respectively. The nickel spectral signal and calibration curve are shown in Figure 4.

Figure 4A shows the absorption spectra of 20 groups standard solutions of nickel ion at different concentrations with 20 g/L zinc as reference. Figure 4B shows the linear correction of nickel at 486 nm. It can be seen that the linear correction curve has a large deviation from the actual measured value, indicating that the nickel linearity is very poor. Figure 4C shows the non-linear calibration curve of nickel at 486 nm, which is fitted by the fourth-order polynomial. It can be seen that the fitting effect is good, indicating that the non-linear calibration curve can well reflect the spectral characteristics of nickel, in which the regression coefficient is 0.9963, and the fitting coefficient is (0.0037, −0.0448, 0.1760, −0.2303, 0.2054).

### C. Extended Kalman Filter Spectrophotometry

**FIGURE 5:**
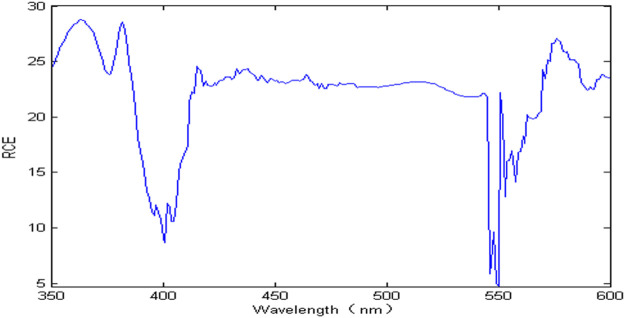
The RCE value of nickel at different wavelengths.

According to nickel spectral characteristics, in the whole wavelength range, the nickel spectral signal completely overlaps with zinc, and the nickel spectra exhibits nonlinearity. When the zinc concentration is oversaturated, its absorbance remains unchanged. The interference of zinc can be eliminated by the difference of spectral signal between the measuring solution and the 20 g/L zinc solution, and then the nickel concentration can be detected iteratively by extended Kalman filter spectrophotometry. In order to improve the accuracy of the EKF model and reduce the calculation time, this paper uses the wavelength selection method based on correlation coefficient to select wavelength variables. The RCE value represents the ratio of correlation coefficient to estimation error, which helps accelerate the convergence speed of filtering and improve the accuracy of the EKF model, shown in Figure 5. It can be seen that the RCE value changes drastically and only stabilizes in the wavelength range of 410–540 nm, which shows that in this wavelength range, the nickel spectral signal has high sensitivity, large amount of information and high correlation coefficient.

**FIGURE 6:**
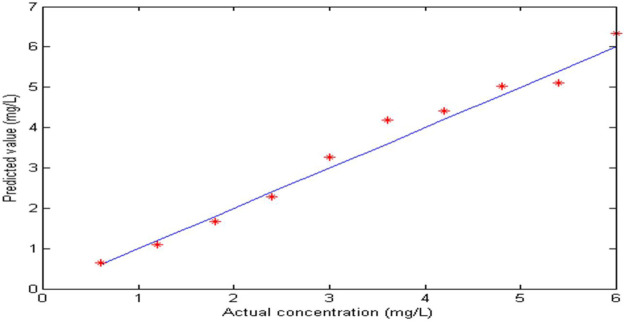
Predicted and actual concentration values of nickel in mixture solution.

In order to evaluate the performance of extended Kalman filter (EKF), 10 groups of nickel and zinc mixed solutions with different concentrations were prepared. The concentration of zinc is 20–30 g/L and that of nickel is 0.6–6 mg/L. During the measurement, a 20 g/L zinc solution is prepared as the reference solution. The extended Kalman filter spectrophotometry (EKF) was used to detect nickel in 10 groups of mixed solutions. The predicted and calibrated values of nickel ion are shown in Figure 6. The maximum prediction relative error of nickel concentration is 16.14%, and the average relative error is 7.28%. The root mean square error of prediction (RMSEP) is 0.2739. The results show that the extended Kalman nonlinear modeling method can be used for the real-time detection of nickel ion in zinc solution, but the detection accuracy needs to be further improved.

### D. Integrated Modeling Based on Adaboost Algorithm

The proposed extended Kalman filter algorithm can detect nickel in zinc sulfate solution, but the relative error varies greatly, and individual sample error even exceed 10%, which can not meet the requirements of industrial detection. The main reasons for this problem are the fluctuation of the matrix zinc, the mutual interference and suppression between multi metal ions, the random noise of measuring instrument and the weak background interference of reagents such as chromogenic agent. In view of the instability of the extended Kalman algorithm model due to the above reasons, it is necessary to make full use of the characteristic wavelength (410–540 nm) of a single sample to minimize the impact of individual wavelength. In this paper, the idea of integrated modeling is applied to the concentration detection of trace nickel in high concentration zinc solution. The integrated modeling based on the Adaboost algorithm takes the extended Kalman filter as the basic sub model. Through the weighted combination of several basic models, the weight of the weak classifier with large classification error rate is reduced, while the weight of the weak classifier with small classification error rate is increased, so as to improve the sample detection accuracy, enhance the stability of the model, and realize the stable detection of trace nickel in high concentration zinc solution.

**FIGURE 7:**
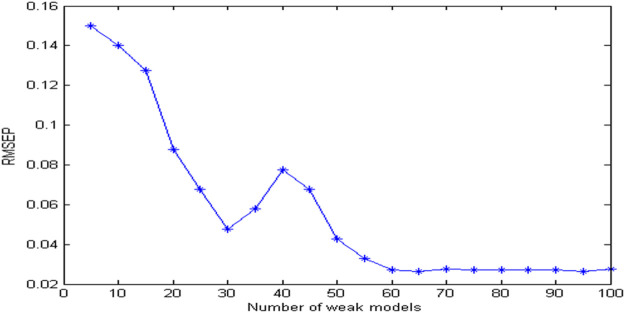
The relationship between the estimation error and the number of weak models.

The more important factor in integrated modeling is the number of weak models. When the number of weak models is too small, the random combination cannot fully cover all the characteristic wavelength information, which affects the accuracy of the results. If the number of weak models is too large, the computational complexity will be increased and the real-time performance of detection will be affected. Figure 7 shows the estimated error graphs using different models. It can be seen from Figure 7 that with the increase of the number of weak models, the estimation error of the model gradually decreases. When the number of weak models is 60, the model gradually tends to be stable, and then increase the number of weak models, the estimation error is basically unchanged. Therefore, the number of weak models is set to 60.

### E. Determination of Nickel by EKF_Adaboost Method

In order to evaluate the performance of the EKF_Adaboost method, ten sets of mixed solutions containing different proportions of Zn and Ni were prepared, and a 20 g/L zinc solution was prepared as a reference. The predicted concentrations of Ni are shown in Table 1. It can be seen that the maximum prediction relative error of nickel is 7.13%, the average relative error is 4.39%, and the root mean square error of prediction (RMSEP) is 0.165. In order to further verify the stability of the EKF_ Adaboost method, 30 repeatability tests were performed on the nickel experimental data, and the PLS, EKF and EKF_Adaboost models were used to detect nickel concentration. The performance comparison results are shown in Table 2.

**TABLE 1:** The predicted concentrations of Ni by EKF_Adaboost method.

No	Actual value (mg/L)		Predicted value (mg/L)	Relative deviation (%)
	Zn	Ni	Ni	Ni
1	2.1 × 104	2.40	2.309	3.79
2	2.2 × 104	4.80	5.002	4.21
3	2.3 × 104	0.60	0.573	4.50
4	2.4 × 104	3.00	3.214	7.13
5	2.5 × 104	5.40	5.653	4.69
6	2.6 × 104	1.20	1.157	3.58
7	2.7 × 104	3.60	3.721	3.36
8	2.8 × 104	6.00	6.237	3.95
9	2.9 × 104	1.80	1.724	4.22
10	3.0 × 104	4.20	4.391	4.54
**Average relative deviation (%)**				**4.39**
**RMSEP**				**0.165**

**TABLE 2:** Comparison of PLS, EKF and EKF_ Adaboost models.

Detection	Evaluation index	PLS	EKF	EKF_ Adaboost
Ni	Maximum relative error	76.57%	19.62%	7.14%
	Average relative error	28.93%	9.38%	4.56%
	RMSEP	0.894	0.329	0.186
	Correlation coefficient	0.5271	0.9942	0.9958
	Detection accuracy	71.07%	90.62%	95.44%

It can be seen from Table 2 that due to the strong nonlinearity between the absorbance and concentration of nickel ion, the performance of using the PLS method to detect nickel concentration is very poor. The maximum relative error is 76.57%, the average relative error is 28.93%, and the detection accuracy is only 71.07%, which can not to meet the requirements of industrial detection. The EKF method uses nonlinear modeling, the average relative error is 9.38%, the detection accuracy is 90.62%, which can better meet the requirements of industrial detection. However, EKF model relies on wavelength variables. When the sample changes due to the random fluctuation of matrix zinc, mutual interference between multi metal ions and random noise of testing instrument, the performance of some wavelength variables of EKF model becomes worse, which will seriously affect the prediction accuracy of nickel ion. EKF_AdaBoost adopts the idea of integrated modeling, which can reduce the dependence on wavelength. The average relative error is 4.56%, and the detection accuracy is 95.44%. Therefore, the EKF_AdaBoost method can realize the real-time detection of impurity nickel ion in zinc sulfate solution.

## Conclusions

In the zinc hydrometallurgical purification process, the concentration ratio of zinc ion to trace nickel ion is as high as 10^5^, and the spectral signal of zinc and nickel overlap seriously, which makes the nickel signal completely masked by the high concentration zinc signal, resulting in the low sensitivity and nonlinear characteristics of the nickel spectral signal. Aiming at the problem that it is difficult to detect nickel in high zinc solution, an extended Kalman nonlinear integrated modeling method based on Adaboost is proposed in this paper to realize the accurate detection of nickel in zinc sulfate solution. The PLS, EKF, and EKF_Adaboost models are used to analyze and detect nickel concentration, and the performance of different models is compared. The results show that EKF_Adaboost adopts the idea of integrated modeling, which can reduce the dependence on wavelength, with an average relative error of 4.56% and a detection accuracy of 95.44%. Therefore, the EKF_Adaboost method can realize real-time detection of impurity nickel concentration in zinc hydrometallurgical purification process, and will be applied to other spectral signals such as infrared spectra, Raman spectra, and more.

## Data Availability

The original contributions presented in the study are included in the article/Supplementary Material, further inquiries can be directed to the corresponding authors.
